# Dynamic changes in PSA levels predict prognostic outcomes in prostate cancer patients undergoing androgen -deprivation therapy: A multicenter retrospective analysis

**DOI:** 10.3389/fonc.2023.1047388

**Published:** 2023-02-09

**Authors:** Mingqiu Hu, Yifeng Mao, Chao Guan, Zhizhong Tang, Zhihang Bao, Yingbang Li, Guowu Liang

**Affiliations:** ^1^ Department of Urology, Maoming People’s Hospital, Maoming, China; ^2^ Department of Urology, the Second Affiliated Hospital of Bengbu Medical College, Bengbu, China; ^3^ Department of Center of science, Maoming People’s Hospital, Guangdong, China; ^4^ Anhui Province Key Laboratory of Translational Cancer Research, Bengbu Medical University, Anhui, China

**Keywords:** prostatic cancer, PSA changes, androgen deprivation treatment, biochemical progress-free survival, time to nadir PSA

## Abstract

**Background:**

Androgen-deprivation therapy (ADT) is used for the treatment of prostate cancer. However, the specific risk factors for the development of castration-resistant disease are still unclear. The present study sought to identify predictors of patient prognostic outcomes through analyses of clinical findings in large numbers of prostate cancer patients following ADT treatment.

**Methods:**

Data pertaining to 163 prostate cancer patients treated at the Second Affiliated Hospital of Bengbu Medical University and Maoming People’s Hospital from January 1, 2015, to December 30, 2020, were retrospectively analyzed. Dynamic changes in prostate-specific antigen (PSA) levels were regularly assessed, including both time to nadir (TTN) and nadir PSA (nPSA). Univariate and multivariate analyses were performed with Cox risk proportional regression models, while differences in biochemical progression-free survival (bPFS) were compared among groups with Kaplan-Meier curves and log-rank tests.

**Results:**

The bPFS values over the median 43.5-month follow-up period differed significantly between patients with nPSA levels < 0.2 ng/mL and ≥ 0.2 ng/mL, being 27.6 months and 13.5 months, respectively (log-rank P < 0.001). A significant difference in median bPFS was also observed when comparing patients with a TTN ≥ 9 months (27.8 months) to those with a TTN < 9 months (13.5 months) (log-rank P < 0.001).

**Conclusions:**

TTN and nPSA are valuable predictors of prognosis in prostate cancer patients after ADT treatment, with better outcomes evident in patients with nPSA < 0.2 ng/mL and TTN > 9 months.

## Introduction

Prostate cancer is an increasingly common cause of human morbidity and mortality. In China, the incidence and mortality of prostate cancer in China account for 8.2% and 13.6% of the global estimates, respectively, according to the GLOBOCAN2020 data (1). As of 2015, prostate cancer was estimated to affect 10 out of every 100,000 people in mainland China, making it the most common urological malignancy (2). Approximately 68% of prostate cancer patients in mainland China have metastatic tumors at the time of diagnosis and androgen deprivation is the standard treatment modality for this type of prostate cancer (3). However, following an initial period during which patients respond well to ADT (median duration of 18-24 months) (4), the levels of prostate-specific antigen (PSA) tend to rise along with disease progression due to the emergence of castration-resistant prostate cancer (CRPC).

The identification of accurate and robust predictive biomarkers associated with prostate cancer patient outcomes following ADT remains an active research hotspot. Since the initial establishment of PSA as a relevant biomarker three decades ago, PSA levels are usually monitored during prostate cancer screening and diagnosis, and also offer value in the context of patient prognostic evaluation (4–6). After the initiation of ADT in patients with prostate cancer, the PSA levels undergo dynamic changes and monitoring these changes can provide insight into the patient outcomes. However, the actual prognostic value of parameters such as the nadir PSA (nPSA) and time to nPSA (TTN) levels remains the subject of substantial debate and further clinical analyses will be critical to fully clarify the relevance of these variables in different patient cohorts (7–9).

Several recent reports have indicated the importance of both nPSA and TNN values as prognostic indicators in the context of ADT treatment. For example, Matsubara et al. reported that an nPSA > 0.1 ng/mL and a TTN of > 6 months were consistent with favorable long-term prognosis (10). Several studies have also demonstrated relationships between these two parameters and the overall survival (OS) or progression-free survival (rPFS) of prostate cancer patients (10–12). However, to our knowledge, there is little information on the value of the PSA halving time in prognostic prediction. Here, a retrospective analysis of patients with prostate cancer patients who underwent ADT treatment was performed to more fully understand the associations between nPSA, TTN, PSA halving time, and patient survival outcomes.

## Methods

### Patient selection

The study conducted a retrospective analysis of 163 patients with prostate cancer ≥ 50 years of age who were treated between January 1, 2015, and December 30, 2020, at two medical centers in China, namely, the Second Affiliated Hospital of Bengbu Medical College and Maoming People’s Hospital. Patients were diagnosed with metastatic or locally advanced prostate cancer based on prostate biopsy analyses and treated with androgen deprivation in the form of total androgen blockade (i.e., surgical/pharmacological castration + anti-androgen drugs). Patients who had been diagnosed with another type of cancer or who had previously undergone radical surgery, chemotherapy, and/or radiotherapy were excluded from the study.

### Data collection

Patient follow-up data were gathered from hospital medical record systems. Traditional computed tomography (CT) scans and bone imaging were used exclusively for baseline staging and subsequent follow-up. Monthly measurements of serum testosterone and PSA levels were performed for the first two years, with testing performed every three months on average after the first two years. The baseline PSA levels at the initiation of ADT, as well as the nPSA, TTN, and PSA half-lives were analyzed. Serum testosterone concentrations were also analyzed to confirm that patients had successfully achieved a castration-like state (< 50 ng/dL). The nPSA was defined as the lowest measured PSA concentration over the course of ADT while TTN was defined as the interval between ADT initiation and nPSA. The PSA halving time indicated the rate of PSA decline following the initiation of ADT.

Biochemical progression was defined as three consecutively elevated PSA test results one week apart with two test results exceeding the 50% nadir. Biochemical progression-free survival (bPFS) was calculated as the interval between the start of ADT and the first measurement of elevated PSA levels associated with biochemical progression.

Tumor load was determined by the presence of at least four bone metastases and at least one metastasis outside the median bone or in the pelvis, or the presence of visceral metastases, as defined by the Chemohormonal Therapy in Metastatic Hormone-Sensitive Prostate Cancer (CHAARTED) study (13).

### Statistical analysis

Continuous data are presented as means ± standard deviation while categorical data including Gleason scores are presented as medians. Continuous data were compared using ANOVA and categorical data with Fisher’s exact test. Univariate and multivariate analyses of patient bPFS were performed using the Cox proportional hazards model, examining the association between this endpoint and variables including age, BMI, Gleason score, staging, post-treatment PSA halving time, metastatic load, nPSA, and TTN values. Cut-off values were determined using receiver operating characteristic (ROC) curves, and bPFS was assessed using Kaplan-Meier curves and log-rank tests. P-values < 0.05 were considered statistically significant. SPSS 27.0.1.0 (IBM, NY, USA) was used for all data analyses.

## Results

### Patient characteristics

The median age of the 163 enrolled patients was 72 years (range:53-89 years; **Table 1**). The majority of the patients (55.2%) had Gleason scores between 8 and 10 at time of diagnosis with 73.0% of the patients having metastatic (M1) disease. Surgical castration was performed in nine cases (5.5%)while the remaining 94.5% of patients instead underwent ADT (3.6 mg once/28 days) in the form of subcutaneous goserelin acetate treatment. All patients received orally administered 50 mg/day of bicalutamide in parallel with the castration treatment. At the start of ADT, the median PSA level of these patients was 98.59 ng/mL while the mean nPSA value was 2.36 ng/mL.

**Table 1:** Baseline characteristics of patients with prostatic cancer.

Variables		No. of patients
Age (y)	53~90	
Mean ± SD	74.36 ± 6.75	
Median	72	
BMI (kg/m^2^)		
Mean ± SD	23.52 ± 3.26	
Median	24.4	
Subgroups of BMI	%	
< 20	18.40	30
20 ~ 25	56.45	92
> 25	25.15	41
Clinic stage		
T1/T2 M0	7.36	12
T3/T4/N1 M0	19.63	32
M1	73.01	119
Gleason score		
≤ 6	3.07	5
3 + 4 = 7	15.95	26
4 + 3 = 7	25.77	42
8 ~ 10	55.21	90
Methods of ADT		
Surgical castration	5.52	9
Medical castration	94.48	154
Prostatic volume (mL)		
Mean ± SD	55.32 ± 23.54	
Median	47.86	
PSA at diagnosis (ng/ml)		
Mean ± SD	126.47 ± 12.36	
Median	98.59	
Nadir PSA (ng/ml)		
Mean ± SD	2.36 ± 7.36	
subgroups		
< 0.2	44.79	73
0.2 ~ 4	33.74	55
> 4	21.47	35
Time to nadir (month)	9.26	
Bone scan		
Positive	73.00 %	119
Negative	27.00 %	44
Metastasis tumor burden ^a^		
low	26.05 %	31
high	73.95 %	88

Abbreviations: BMI, body mass index; ADT, androgen deprivation treatment; PSA, prostate-specific antigen.

^a^ only including 119 metastasis cases.

### Screening for bPFS predictors in patients with prostate cancer

To define the risk factors associated with bPFS in patients with metastatic prostate cancer, the data of 199 patients were analyzed using univariate and multivariate Cox regression. A decline in the PSA level to ≤ 2 ng/mL (hazard ratio [HR] 0.462, P = 0.001) and a TTN < 9 months (HR 1.736, P = 0.021) were found to be independently associated with the risk of bPFS (**Table 2**). PSA levels < 0.2 ng/ml were associated with favorable prognosis, and the risk of disease progression declined as the time taken to reach the PSA nadir increased.

**Table 2:** Univariate and multivariable analysis of potential risk factors for biochemical progression-free survival.

Variable	Univariate		Multivariate		
	HR (95% CI)	p	HR (95% CI)	p	
Age ^b^	0.965 (0.953~0.992)	0.010	0.993 (0.963~1.023)	0.568	
BMI	1.025 (0.967~1.078)	0.259			
Gleason score	1.146 (0.980~1.354)	0.059			
Stage ^b^	1.452 (1.142~1.698)	0.024	0.986 (0.968~1.028)	0.924	
Metastasis tumor burden ^a, b^	1.534 (1.283~1.845)	0.000	1.152 (0.946~1.521)	0.178	
PSA level at diagnosis ^b^	1.023 (1.001~1.030)	0.002	1.002 (0.989~1.021)	0.910	
PSA nadir (ng/mL)					
> 2	1.000 (reference)		1.000 (reference)		
≤ 2	0.462 (0.374 ~ 0.523)	0.001	0.492 (0.348 ~ 0.583)	0.003	
TTN (months)					
> 9	1.000 (reference)		1.000 (reference)		
≤ 9	1.736 (1.422 ~ 2.033)	0.021	1.713 (1.583~ 2.198)	0.012	
PSA half time (months)					
≤ 3	1.000 (reference)		1.000 (reference)		
> 3	1.346 (1.135 ~ 1.864)	0.338			

CI, confidence interval; BMI, body mass index; ADT, androgen deprivation treatment; PSA, prostate-specific antigen; TTN, time to PSA nadir.

a only including 119 metastasis cases;

b significant at univariate analysis and carried onward to multivariate analysis.

### The relationship between dynamic changes in PSA concentrations and patient bPFS

Lower nPSA levels were found to be associated with a lower risk of bPFS (**Table 3**), and this predictive relationship remained true when controlling for variables including age, BMI, tumor load, baseline PSA level, stage, and Gleason score. For patients with PSA levels < 4 ng/mL (defined as normalization), the odds of PFS were reduced with shorter times to normalization, although no significant association with the emergence of castration resistance was detected (**Table 4**). When intergroup comparisons were performed for patients with normalization times of 8-12, 12-24, or > 24 weeks, it was found that better prognosis was associated with shorter normalization times, with a normalization time > 24 weeks found to be an independent predictor of patient outcome (HR 0.695, P = 0.001). These data indicate that PSA levels that fall more rapidly to within the reference ranges following ADT initiation are associated with better patient outcomes. Unlike the PSA normalization time, TTN remained a significant predictor of patient bPFS even after controlling for other risk factors and using the cut-off values of 6, 10, or 12 months.

**Table 3:** Cox regression analysis of the relationship between PSA nadir and bPFS.

PSA nadir (ng/mL)	HR (95% CI)	p-value
Univariate analysis		
≤ 0.2	1.000 (reference)	
0.2~1.0	1.329 (1.131 ~ 1.344)	0.014
1.0~2.0	1.921 (1.244 ~ 2.336)	0.016
>2.0	2.538 (1.964 ~ 3.756)	0.000
	p for trend < 0.01	
Multivariate analysis		
≤ 0.2	1.000 (reference)	
0.2~1.0	1.237 (1.190 ~ 1.463)	0.000
1.0~2.0	2.327 (2.153 ~ 3.161)	0.000
>2.0	3.489 (2.268 ~ 3.997)	0.000
	p for trend < 0.01	

PSA, prostate-specific antigen; bPFS, biochemical progression-free survival; HR, hazard ratio; CI, confidence interval.

a only including 11 metastasis cases.

**Table 4:** Cox regression analysis of the relationship between the PSA halving time and bPFS.

	Univariate analysis		Multivariate analysis	
	HR (95 % CI)	p-value	HR (95 % CI)	p-value
Time to PSA ≤ 4 ng/mL (weeks)				
≤ 4	1.000 (reference)		1.000 (reference)	
4~8	0.845 (0.803 ~ 0.948)	0.927		
8~12	0.748 (0.526 ~ 1.048)	0.086		
12~24	0.756 (0.532 ~ 1.067)	0.114		
>24	0.656 (0.476 ~ 0.927)	0.021	0.695 (0.623 ~ 0.821)	0.001
Time to PSA nadir (months)				
≤ 6	1.000 (reference)		1.000 (reference)	
6~9 ^a^	0.936 (0.824 ~ 1.002)	0.026	0.902 (0.824 ~ 0.968)	0.001
> 9 ^a^	0.532 (0.282 ~ 0.713)	0.000	0.503 (0.213 ~ 0.698)	0.000
	p for trend < 0.01		p for trend < 0.01	
PSA halving time (months)				
≤ 0.5	1.000 (reference)		1.000 (reference)	
0.5~1	1.136 (0.995 ~ 1.226)	0.132		
1~1.5	0.910 (0.736 ~ 1.623)	0.364		
1.5~3	0.653 (0.497~ 0.936)	0.098		
> 3	0.536 (0.485 ~ 0.663)	0.063		

a significant at univariate analysis and carried onward to multivariate analysis;

PSA, prostate-specific antigen; bPFS, biochemical progression-free survival.

Regarding the PSA halving time, a shorter halving time was found to be associated with worse prognosis when comparing groups using the cut-off values of 0.5, 1, 1.5, and 3 months. Although a trend towards a worse prognosis with lower PSA halving time wa observed, but it the the group comparisons were not statistically significant. From this we conclude that PSA halving time is not a good predictor of ADT initiation.

**Figure 1:**
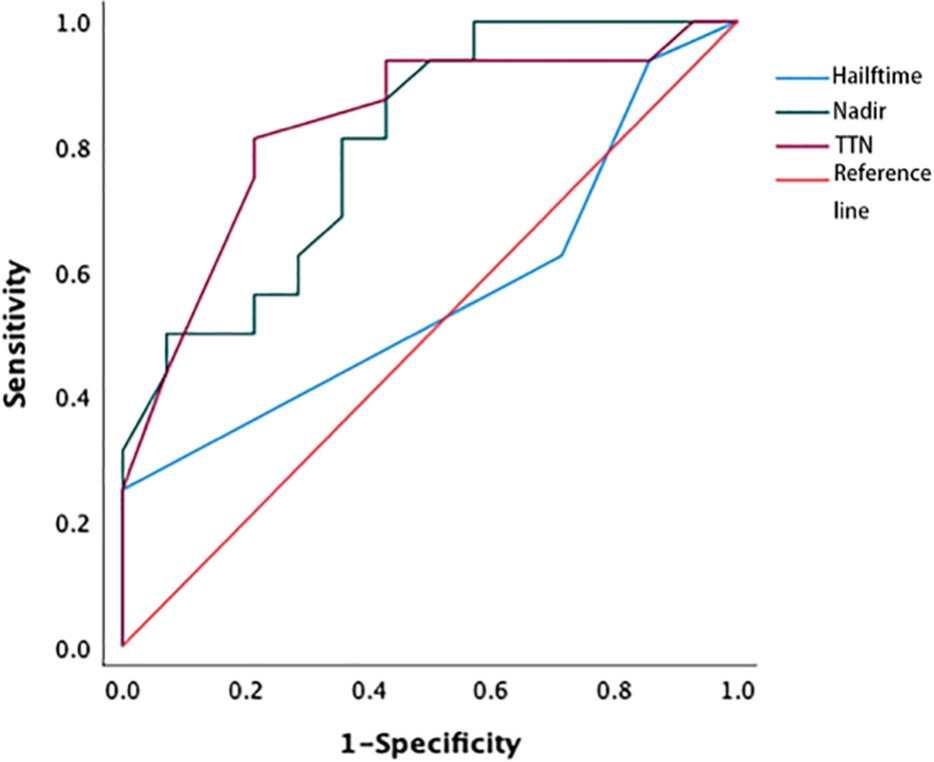
ROC curve of nPSA, TTN, and PSA halving time. The areas under the curve for the PSA nadir and PSA time to nadir were 0.804 and 0.833, respectively, while AUC for the PSA halving time was 0.563. ROC, receiver operating characteristic; TTN, time to PSA nadir.

The area under the curve (AUC) values for nPSA and TTN were 0.804 and 0.833, respectively (**Figure 1**); as these values fall above 0.7 but below 0.9, this suggests that these variables offer some level of prognostic value. The nPSA value was associated with a sensitivity and specificity of 65.7% and 73.6%, respectively, when selecting 0.2 ng/mL as the optimal cut-off value, indicating that nPSA values > 0.2 ng/mL are suggestive of a poorer prognosis. Similarly, 9 months was selected as the optimal TTN cut-off value, yielding a sensitivity and specificity of 71.6% and 73.9%, respectively. Thus, patients with a TTN > 9 months showed better prognostic outcomes. The AUC for the PSA halving time was only 0.563, indicating that this parameter was insufficiently accurate for the reliable assessment of patient bPFS risk.

**Figure 2:**
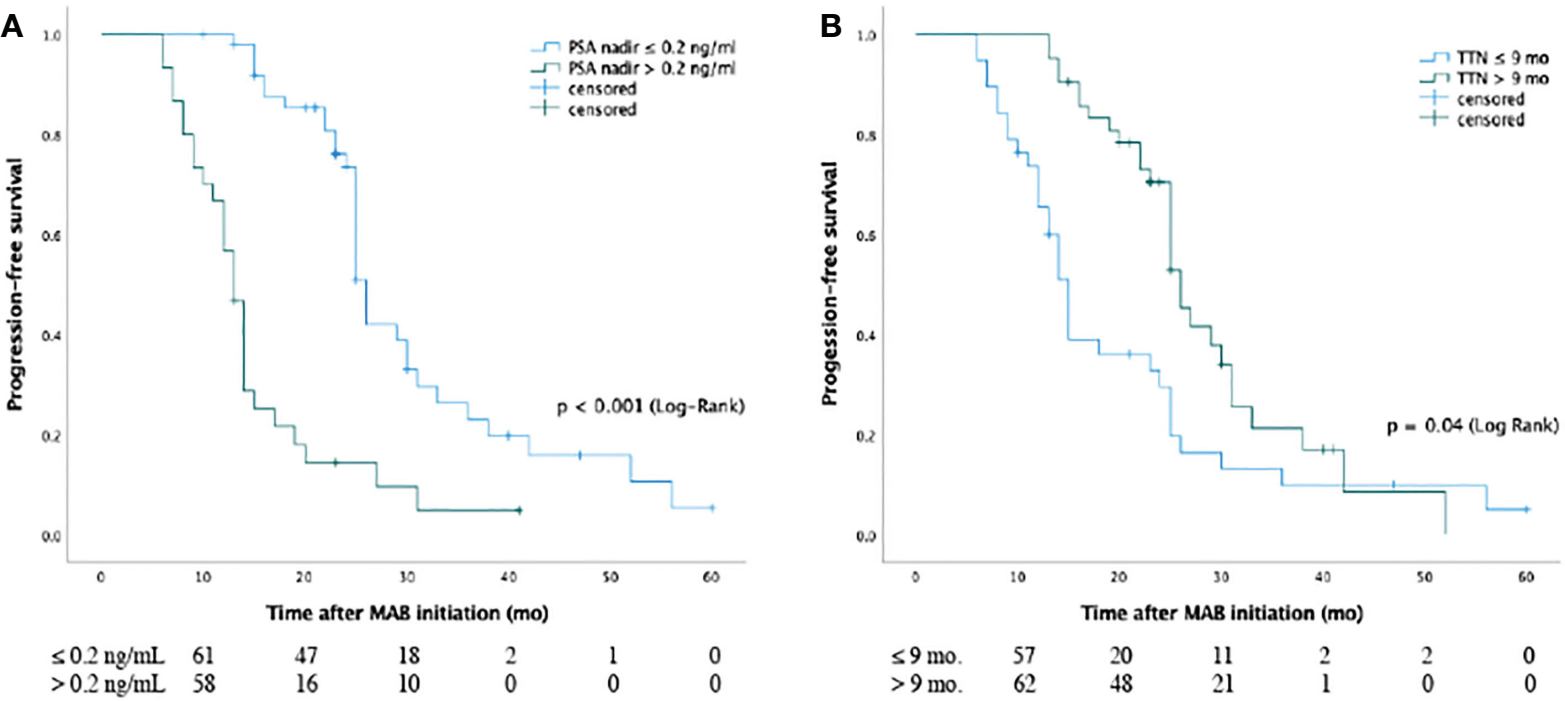
Kaplan-Meier curves for biochemical progression-free survival of nPSA **(A)** and TTN **(B)** for patients with prostate cancer from MAB initiation. The median bPFS values **(A)** were 27.6 months and 13.5 months for patients in the two groups with PSA nadir < 0.2 ng/ml and ≥ 0.2 ng/ml, respectively, and there was significant difference between groups (P < 0.001 [log-rank]). The median bPFS values **(B)** were 27.8 months and 13.5 months for cases in two groups with TTN ≥ 9 months and < 9 months, respectively, and there was significant difference between the two groups (P < 0.001 [log rank]). Abbreviations: PSA: Prostate-specific antigen; TTN: time to PSA nadir; MAB: maximal androgen blockade; nPSA: PSA nadir.

In patients with an nPSA < 0.2 and ≥ 0.2 ng/mL, the respective median bPFS values were 27.6 months and 13.5 months, with a significant difference between these groups (Log-rank P < 0.001). Similarly, patients with a TTN ≥ 9 months showed a better median bPFS (27.8 months) relative to patients with a TTN < 9 months (13.5 months; Log-rank P < 0.001)(**Figure 2**).

## Discussion

Given the high rates of advanced prostate cancer in China, ADT remains an important focus of clinical research interest (9). In addition to aiding the screening and diagnosis of prostate cancer, the PSA level is an invaluable tool to monitor and evaluate the prognosis of patients after the initiation of ADT treatment. Dynamic shifts in PSA concentrations over the course of the treatment can provide insight into patient outcomes, with several variables including nPSA, TTN, and the PSA halving time shown to offer prognostic utility in certain patient populations (12, 14, 15). However, these findings are not universal, and the clinical significance of these PSA dynamics as biomarkers of CRPC development thus remains controversial (8, 16).

Here, it was found that lower nPSA and longer TTN values were significantly associated with better outcomes in patients with prostate cancer. This is consistent with data from several reports demonstrating the prognostic utility of these two variables in patients treated with ADT (17, 18). Shi et al., for example, performed a retrospective analysis of data from 153 individuals with metastatic CRPC undergoing combination ADT and docetaxel treatment, revealing a reduced response to chemotherapy and poorer prognosis in patients with higher nPSA levels and shorter TTN (8). A double-blind randomized trial in which the efficacy of abiraterone acetate and prednisone (AAP) + ADT was compared to placebo + ADT showed that patients with lower nPSA values (0.1 ng/ml) six months after treatment had better prognosis (10). In addition, Choueiri et al. reported that TTN <6 months and an nPSA value of 0.2 ng/mL were independently predictive of shorter OS (19). Harshman et al. suggested that a PSA level of 0.2 ng/mL after seven months of treatment extends the OS of patients with metastatic hormone-sensitive prostate cancer (20). Matsubara et al. found that low PSA levels (0.1 ng/ml) after six months may indicate a good long-term response to treatment (10) while a PSA level of 4 ng/mL or less after seven months was found to be a strong predictor of survival (20). While these prior studies have emphasized the utility of nPSA and TTN values as predictors of OS and PFS in patients with advanced prostate cancer, the optimal predictors differed (14, 19, 21). Our analysis indicated that a PSA < 0.2 ng/mL and a TTN of > 9 months were optimal predictors of bPFS in patients with metastatic prostate.

The present multicenter retrospective case review approach revealed a significant association between nPSA levels < 0.2 ng/mL of nPSA and better median bPFS outcomes, relative to patients with nPSA levels ≥ 0.2 ng/mL of nPSA(27.6 vs 13.5 months, respectively), with these patients showing an overall 67.7% lower progression risk (HR, 0.323). Other studies have similarly reported lower nPSA levels to be related to a better prognosis (9, 22), although the actual cut-off values for nPSA have varied among studies (8, 9, 22–24). Several studies have used a cut-off value of 0.2 ng/mL (8, 23, 25), although some reports suggest that an undetectable nPSA level is related to better prognostic outcomes (26) and other reports have used a higher cut-off of 4.0 ng/mL (22). These variations may be attributable to different follow-up approaches and study population characteristics.

It is generally believed that a more rapid decline in the PSA level is associated with a greater likelihood of eliminating the proportion of reactive prostate cancer cells, leading to increased patient survival. However, it was found that while rapidly declining PSA levels and the transcriptional outcome of ADT were related, they were not associated with cancer cell death. Alternatively, a rapid decrease in PSA levels may indicate the downregulation of PSA expression in hormone-sensitive prostate cancer cells as PSA expression is regulated by androgen through the androgen receptor pathway (6, 12). In this study, TTN was found to be associated with prognosis in prostate cancer patients, with a longer TTN being similarly associated with a longer bPFS. Specifically, patients with a TTN > 9 months exhibited a longer median survival than did patients with a TTN < 9 months (27.8 vs 13.5 months; *P* = 0.004), in line with prior reports (23, 24, 27–29). Different studies have used TTN cut-off values from 6-12 months, with a cut-off of 9 months being the most common (24, 27).

Here, lower nPSA values were associated with lower odds of bPFS (*P* < 0.001), consistent with prior reports (30). This study performed subgroup analyses by treating nPSA and TTN as continuous variables. The results showed that the likelihood of bPFS increased 1.794-fold in the subgroup with nPSA levels between 0.2 and 4 ng/mL, and 5.332-fold in the subgroup with nPSA levels > 4 ng/mL, compared to the subgroup with TTN > 12 months, and that the risk increased by 1.245-fold at TTN < 12 months and 3.408-fold at TTN < 6 months compared to the subgroup with TTN > 12 months (30).

Here, ROC curves revealed that the PSA halving time was unrelated to prognostic outcomes in patients undergoing ADT, with an AUC of just 0.553. This may be attributable to the fact that reductions in PSA levels following the initiation of ADT do not occur at a constant rate. Indeed, previous findings suggest that the response of prostate cancer to ADT is triphasic, including an androgen-PSA response, tumor atrophy, and quiescent proliferation phases (31). Given that prostate tumors are heterogeneous, they are likely to harbor a proportion of hormone-unresponsive cells leading to inevitable differences in the rates at which the PSA levels decline and tumors adapt to ADT treatment. Accordingly, the PSA halving time cannot reliably reflect the proportions of androgen-independent cells in a given patient, rendering it unsuitable for prognostic evaluation in prostate cancer patients (14).

Early PSA responses to ADT (>30% reduction after 4 or 12 weeks) are frequently used to gauge patient prognosis (32, 33). Dynamic shifts in serum PSA concentrations have been used as tools both to explore the prognosis of hormone-sensitive prostate cancer patients and monitor CRPC patients undergoing second-line ADT (34, 35) while also providing an approach for assessing the prognosis of CRPC patients undergoing chemotherapy (36).

There are several limitations to this study. These include the relatively small sample size, retrospective design, and the potential for differences in evaluation criteria at the two medical centers. In addition, other common measures of patient survival such as OS or disease-free survival were not analyzed. Moreover, the current standard of care for patients diagnosed with metastatic hormone-sensitive prostate cancer besides ADT, including Abirateone or AR blocking (Enzalutamide/apalutamide/daralotamide+docetaxel), was not included in the statistics.

## Conclusions

These results suggest that both TTN and nPSA offer value as predictors of prostate cancer patient outcomes following ADT, with better outcomes seen in patients with an nPSA level < 0.2 ng/mL and a TTN > 9 months. These findings provide a foundation for guiding the treatment and monitoring of prostate cancer patients.

## Data availability statement

The original contributions presented in the study are included in the article/supplementary material. Further inquiries can be directed to the corresponding author.

## Author contributions

Conceptualization, MH, CG, ZT; methodology, MH and YM; software, YM; validation, MH, YM and ZB; investigation, YM; resources, ZB, YL and GL; data curation, YL and GL; writing—original draft preparation, YM, ZB; writing—review and editing, MH and YM; visualization, YM; supervision, MH, CG, ZT; project administration, MH, CG, ZT. All authors have read and agreed to the published version of the manuscript.
